# Hybrid operating rooms for the treatment of complex middle cerebral artery aneurysms: A technical note

**DOI:** 10.1016/j.bas.2026.105934

**Published:** 2026-02-17

**Authors:** Dieter Thijs, Ana Rodríguez-Hernánde, Tomas Menovsky, Hieronymus Damianus Boogaarts

**Affiliations:** a Department of Neurosurgery, Antwerp University Hospital, Edegem, Belgium; b Department of Neurological Surgery Germans Trias I Pujol University Hospital Universidad Autónoma, Barcelona, Spain; c Department of Neurosurgery, Radboud University Medical Center, Nijmegen, the Netherlands

**Keywords:** Middle cerebral artery aneurysm, Complex aneurysms, Hybrid operating room, Flash fluorescence, EC-IC bypass

## Abstract

**Background:**

The recent emergence of hybrid operating rooms (H-OR) broadens treatment options for complex intracranial aneurysms. Combining endovascular options enabled by the H-OR with refined microsurgical techniques could provide less arduous treatment alternatives for large, distal, and fusiform MCA aneurysms.

**Research question:**

To describe the technical nuances of a hybrid treatment consisting of microcatheter-guided flash fluorescence to enable revascularization bypass and endovascular parent vessel occlusion (PVO) in three different cases of large, distal and fusiform MCA aneurysms.

**Methods:**

A technical report of three cases is provided. At a H-OR, after STA harvest and side-appropriate craniotomy, femoral artery access was obtained and a microcatheter was guided in the corresponding aneurysm. Indocyanine green (ICG) was injected throughout the microcatheter while running a videoangiography on the surgical microscope, allowing straight-forward identification of the appropriate vessel recipient for the revascularization bypass.

**Results:**

The described hybrid flash-fluorescence technique allowed straight-forward identification of the appropriate vessel recipient for the revascularization bypass in all three cases. After completion of the bypass and verification of its patency by intraoperative angiography, the aneurysms and their parent vessels were occluded endovascularly.

**Conclusion:**

This hybrid treatment using modern endovascular and microsurgical techniques obviates the need for a large craniotomy, Sylvian fissure dissection and aneurysm manipulation, while reducing surgical risks and operative time in the treatment of complex fusiform MCA aneurysms. The H-OR room can result in new proficient, creative and safe treatment options of different neurovascular pathologies.

## HIGHLIGHTS

• Hybrid operating rooms broaden treatment options for complex intracranial aneurysms
• Microcatheter-guided flash fluorescence enabled easier revascularization bypass
• Combined with endovascular, provided new alternatives for fusiform MCA aneurysms
• Further availability of hybrid operating rooms may enable new creative treatments

## 1 Introduction

Treatment of large, distal and fusiform middle cerebral artery (MCA) aneurysms is technically demanding and often requires a different treatment strategy compared to classical saccular aneurysms. Open surgical reconstruction of the diseased segment with direct flow preservation or revascularization bypass is often needed (Wessels et al., 2019). However, any of the features of a complex MCA aneurysm can pose a challenge to those surgical options. Large size, distal location and fusiform morphology, alone or in combination, may prevent easy dissection/identification of parent vessel and aneurysm's outflow and may place the lesion in narrow operative corridors requiring extensive sylvian fissure split through eloquent brain areas. Furthermore, when a revascularization bypass is needed, anastomosis to deep seated efferent branches can be difficult, and the alternative of identifying an appropriate cortical recipient artery may not be straightforward.

Taking advantage of intraoperative technologies such as the indocyanine green videoangiography (ICG-VA), new microsurgical techniques to overcome these challenges have been developed (Raabe et al., 2003). For example, any of the different described variations of the so-called Flash Fluorescence Technique, help avoid extensive subarachnoid dissection and enable the identification of cortical recipient arteries for a less demanding and technically easier bypass (Esposito et al., 2012; Rodriguez-Hernandez et al., 2012; Yokota et al., 2017).

Interestingly, the recent emergence of hybrid operating rooms (H-OR) may offer even broader possibilities in this arena. By building upon previous microsurgery advances such as the aforementioned flash fluorescence, and combining them with the high-end endovascular modalities enabled by the H-OR, successful treatment of complex MCA aneurysms may become less arduous. Our study aims to portray the usefulness of a H-OR for the treatment of three large and fusiform MCA aneurysms, and aims to describe the technical nuances of a hybrid technique consisting of microcatheter-guided flash fluorescence to enable revascularization bypass and endovascular parent vessel occlusion (PVO).

## 2 Methods

For this technical report based on three clinical cases, Institutional Review Board approval was waived. All three patients consented to the publication of their diagnostic images and clinical details, yet they have all been anonymized.

### 2.1 Case presentations

**Fig. 1:**
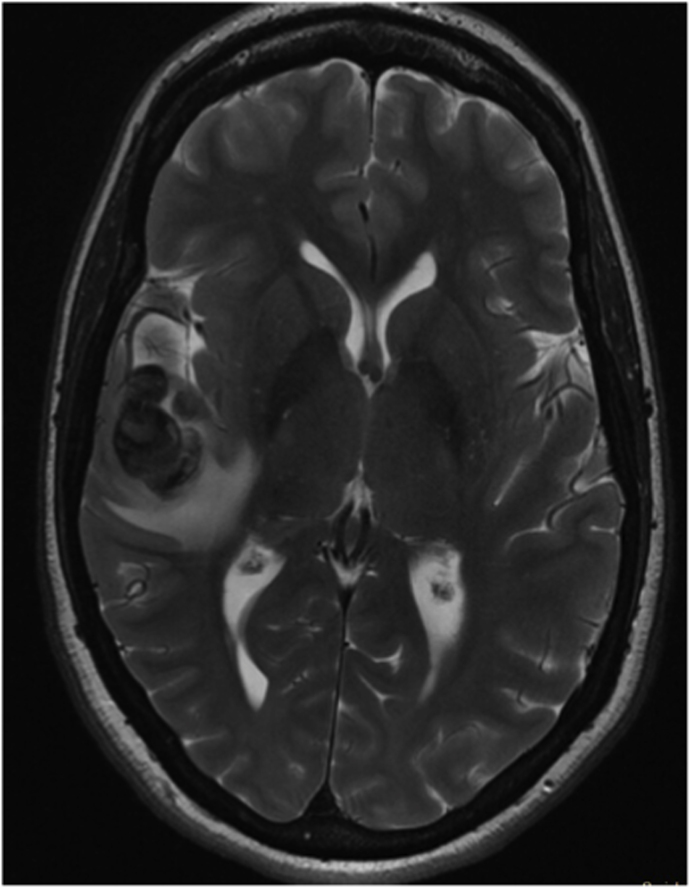

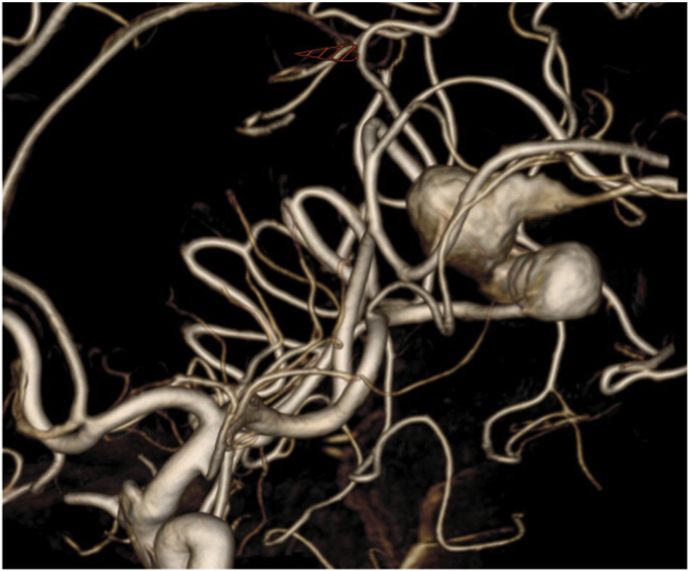
***Diagnostic Images of Case #1*. A)** T2 weighted brain MRI shows a large distal middle cerebral artery aneurysm with perilesional edema. **B** Digital Subtraction Angiography; 3D Reconstruction of internal carotid artery injection confirming the fusiform lobulated aneurysm and showing the exiting M3 branch.

The first case was a patient in their early 30s who presented with a short episode of sensory aphasia possibly related to a focal epileptic seizure. This episode was preceded by headache and tinnitus a few days earlier. Computed tomography (CT), CT angiography, and magnetic resonance imaging (MRI) of the brain revealed a large, 21 mm fusiform MCA aneurysm on a right M3 branch with surrounding edema (Fig. 1A). Digital subtraction angiography (DSA) confirmed a large, partially thrombosed M3 aneurysm with a post-dilation tract of such M3 branch (Fig. 1B).

**Fig. 2:**
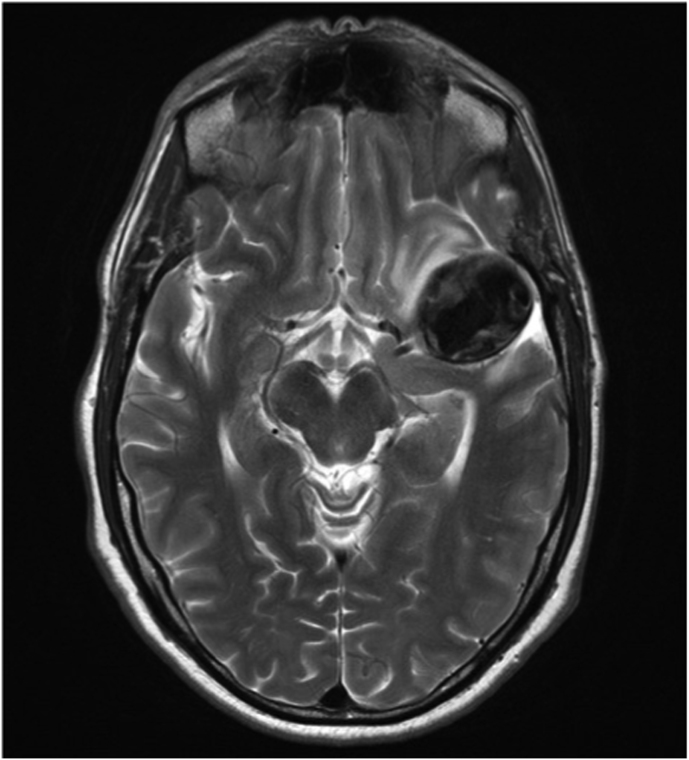

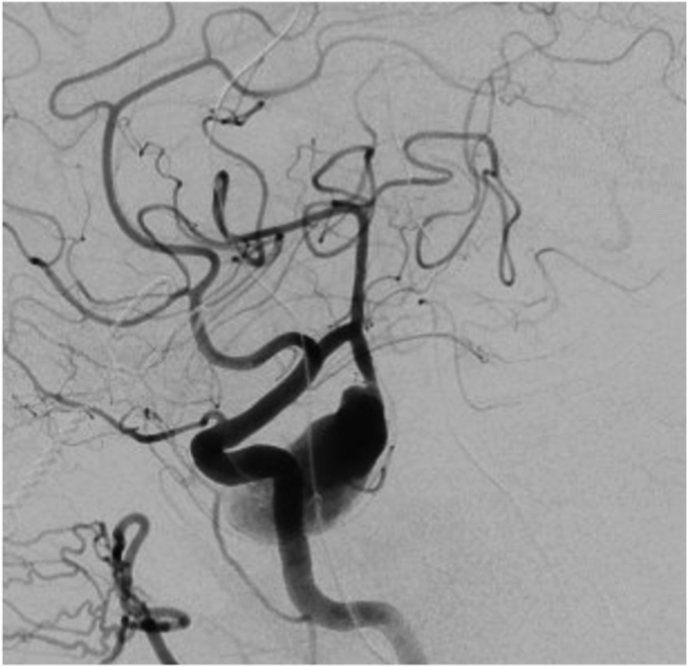
***Diagnostic Images of Case #2.* A)** Diagnostic MRI showing the partially thrombosed distal MCA aneurysm. **B** Digital Subtraction Angiography, left ICA injection, lateral view showing the large fusiform aneurysm.

**Fig. 3:**
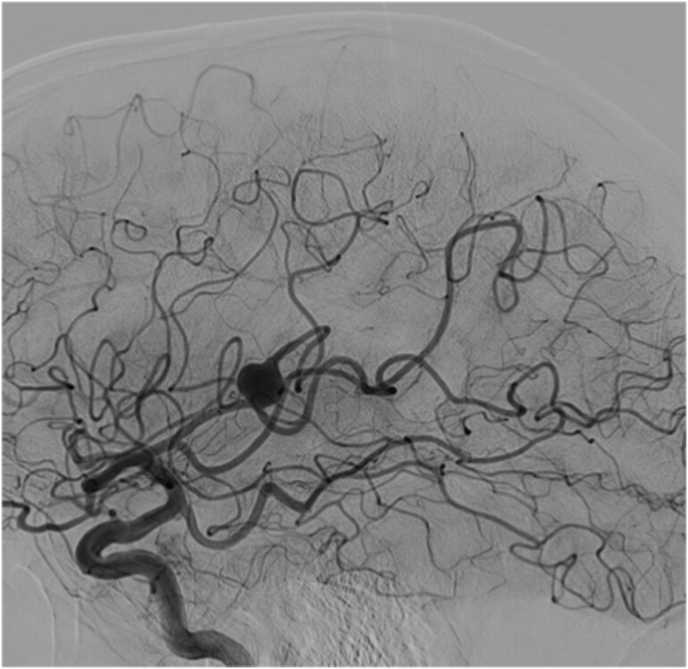

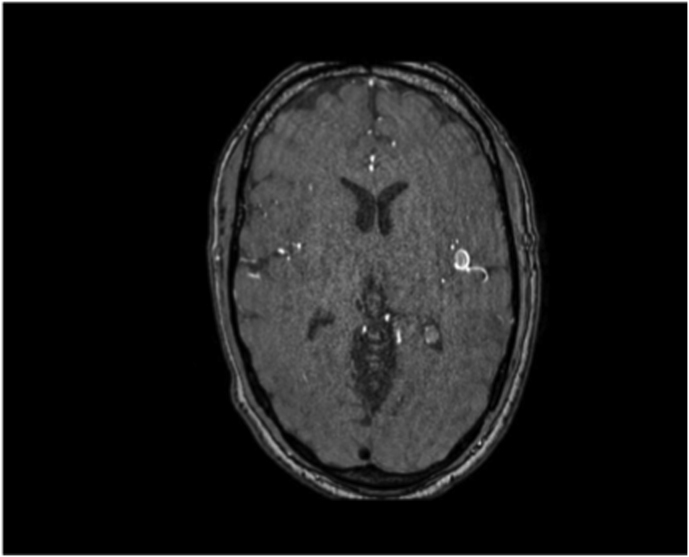
***Diagnostic Images of Case #3***. **A** Diagnostic DSA, ICA injection, lateral view showing the distal fusiform MCA aneurysm. B**)** Diagnostic MRI depicting the deep location of the aneurysm and the efferent branches within the distal Sylvian fissure.

Case 2 was a patient in their 60s who presented to our emergency room with a seizure. Brain MRI revealed a partially thrombosed MCA aneurysm with surrounding edema and no signs of hemorrhage (Fig. 2A). DSA showed a large, partially thrombosed M2 aneurysm with a dysplastic distal M2 segment (Fig. 2B). Case 3 was a patient in their 40s who was diagnosed elsewhere with an incidental left distal MCA aneurysm. Upon detecting significant size growth on follow-up image, the patient was referred to our clinic for treatment. DSA revealed a 9 mm M3 aneurysm with fusiform morphology (Fig. 3A) distally located within the left Sylvian fissure as shown by the MRI (Fig. 3A). An overview of the relevant patient data and treatment characteristics is provided in Table 1.

**Table 1:** Patient characteristics.

CASE NUMBER	AGE (years)	GENDER (F/M)	PRESENTATION	ANEURYSM SIZE	ANEURYSM MORPHOLOGY	TREATMENT DETAILS	FOLLOW-UP DURATION (months)
1	35	F	Sensory aphasia	21 mm	Fusiform/M3 branch	Right STA-MCA bypass + coil occlusion of aneurysm	32
2	64	M	Epileptic Seizure	44 mm	Partially Thrombosed/M2	Left STA-MCA bypass + coil occlusion of aneurysm	21
3	43	F	Incidental	9 mm	Fusiform/M3 branch	Left STA-MCA bypass + coil occlusion of aneurysm	12

### 2.2 Management in the hybrid operating room

In all three cases, flow diverter stenting or surgical clip reconstruction were deemed inferior to trapping and exclusion of the diseased arterial segment. Thus, a minimally invasive hybrid treatment option was devised combining microsurgical and endovascular techniques. The plan consisted of endovascular parent vessel occlusion (PVO) preceded by flow preservation using an STA to MCA bypass.

The cases were performed at a hybrid operating room equipped with a multi-axial uniplanar angiograph (ARTIS pheno, Siemens Healthcare GmbH, Erlangen, Germany) that enables diagnostic angiograms, 3-D reconstruction images and endovascular treatment.

**Fig. 4:**
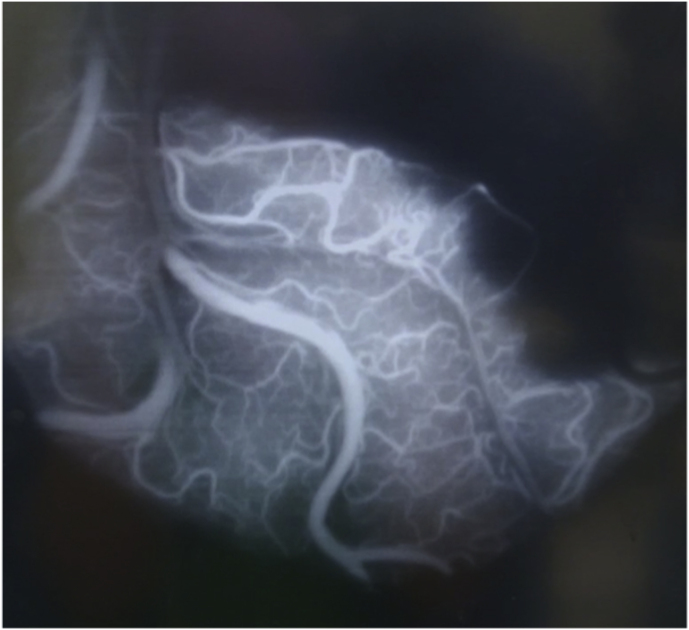

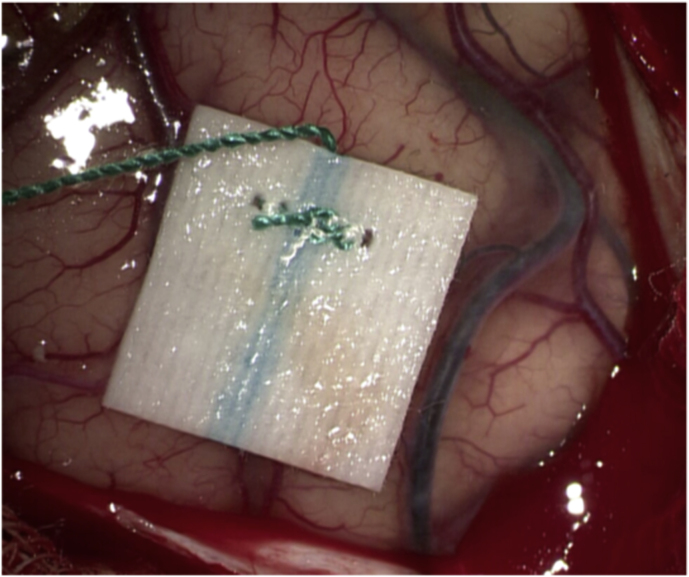

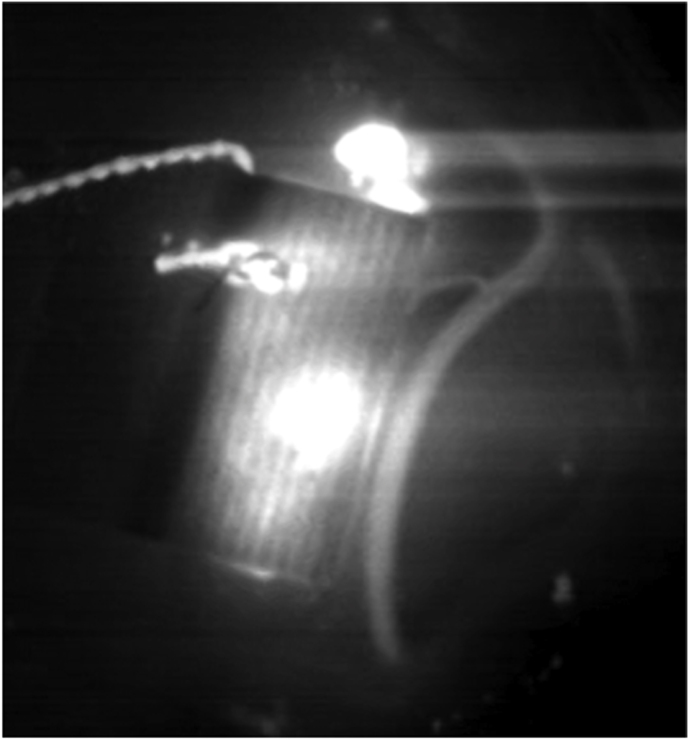

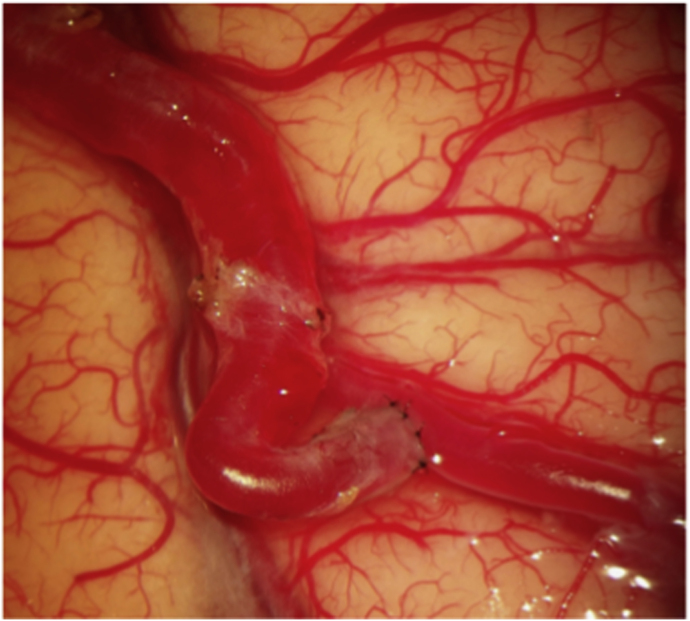
**Intraoperative Images of “Hybrid Flash Fluorescence”. A)***Intraoperative image from case #2.* Superselective Hybrid ICG-videoangiography to select the cortical recipient vessel for the bypass. **B)** and **C)***Intraoperative images from case #3.***S**uperselective hybrid injection of ICG identifying the bypass' recipient vessel both by visual inspection (note the green color) and by “hybrid fluorescence” with videoangiography. **D)***Intraoperative image* from case #2 showing the end-to-side STA-MCA anastomosis.

**Fig. 5:**
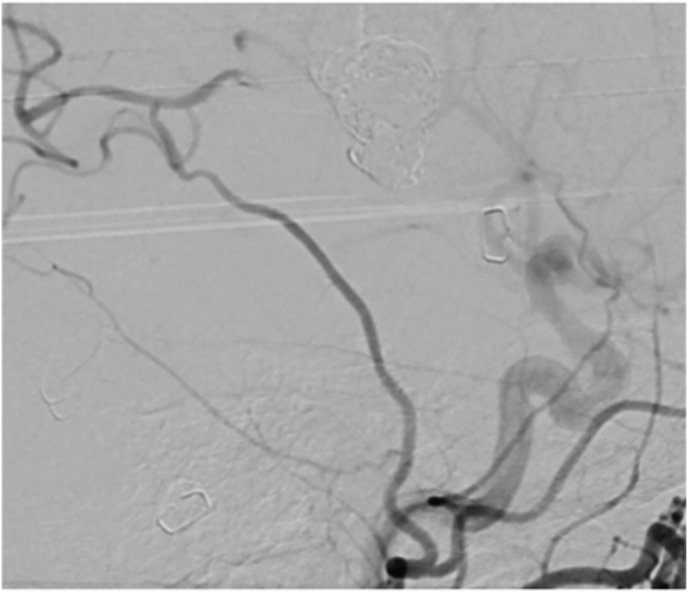

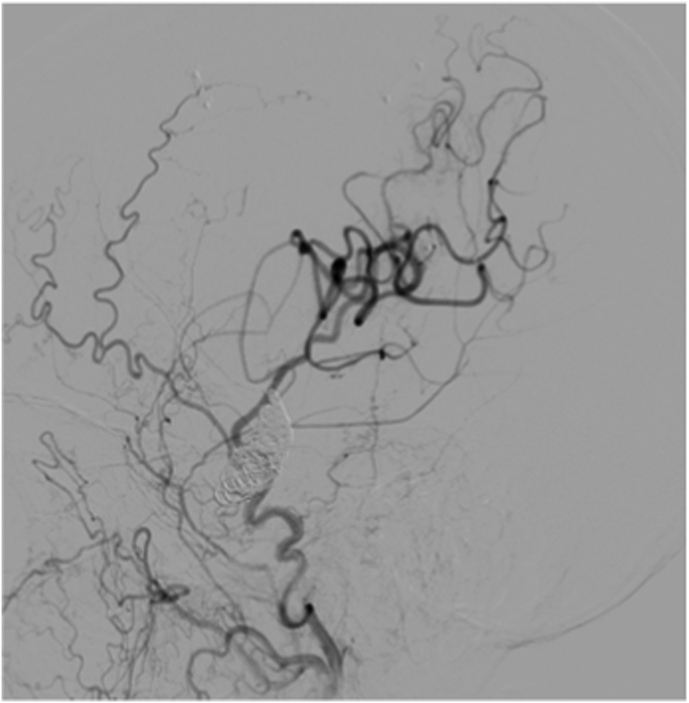

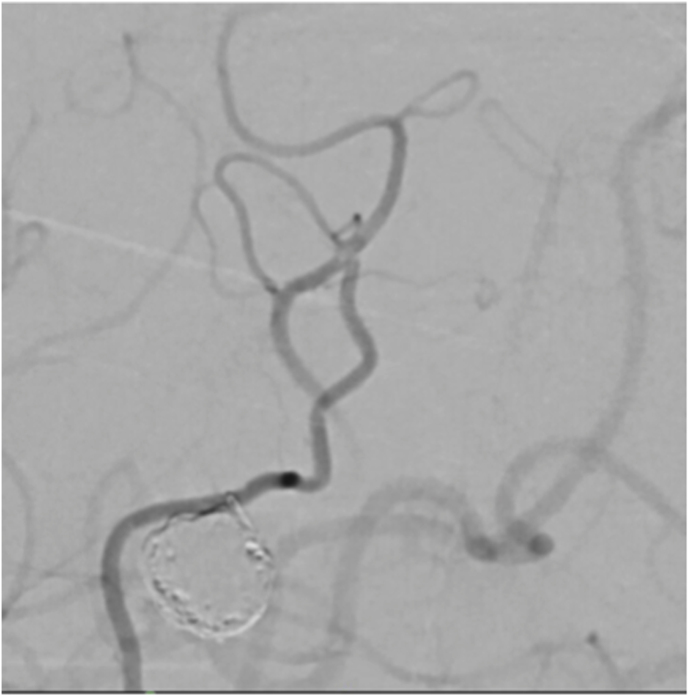
**Aneurysms occlusion and distal revascularization**. *Intraoperative DSA (ECA injection) from****A)****case #1;****B)****case #2; and****C)****case #3*, showing aneurysm occlusion with coils, bypass patency, and revascularization of distal MCA territory.

Patients were pretreated with acetylsalicylic acid (ASA) 100 mg daily during the week preceding surgery. Under general anesthesia, the right STA for case #1 and the left STA for case #2 and case #3 were carefully harvested and side-appropriate fronto-temporal craniotomies were performed in each patient. Next, right femoral artery access was obtained and a 6 french Envoy guider was used for 45° SL 10 microcatheter. The microcatheter was guided in the corresponding aneurysm. Indocyanine green (ICG) was injected into the aneurysms while performing a video angiography (VA) on a Zeiss Pentero (Carl Zeiss Meditec, Jena, Germany) surgical microscope. Direct flash fluorescence on the cortical surface clearly identified the appropriate cortical branch recipient for the revascularization bypasses (Fig. 4A, B and 4C)). An end-to-side STA-MCA anastomosis was then performed on the identified artery using interrupted 10-0 nylon sutures (Fig. 4D). After confirmation of the bypass patency with intraoperative angiography, the aneurysm was occluded endovascularly with microcoils. In all three cases, the aneurysm was completely occluded, resulting in intentional closure of the parent vessel. Care must be taken to ensure that coils do not protrude into the parent artery, either before or beyond the aneurysm, as this could endanger small branch vessels and lead to their occlusion. Intraoperative external carotid artery angiographic injection demonstrated filling of the distal territory of the occluded parent vessel through the STA-MCA bypass in every case. (Fig. 5A, B, C).

## 3 Results

First two patients made an uneventful recovery with no new neurological deficits and were discharged home on day 3 after the hybrid procedure. Follow up MRI of patient 1 showed resolution of the peri-aneurysmal edema. Patient #3 presented a mild expressive postoperative aphasia that recovered completely within the first week. CT showed slight cortical swelling, CTA imaging showed no abnormalities. At last available follow-up (32 months, 21 months and 12 months respectively), all three patients showed complete aneurysm occlusion and bypass patency, and remain neurologically asymptomatic.

## 4 Discussion

As illustrated by our three cases, H-ORs offer new possibilities to refine and improve management strategies of complex intracranial aneurysms. The description of the flash fluorescence technique helped simplify the surgical technique for large, distal or fusiform MCA aneurysms. This new “hybrid flash fluorescence” represents one step forward in such cases, that, furthermore, can benefit from a concomitant endovascular treatment.

Large, distal and fusiform MCA aneurysms are rarely amenable to simple clipping or even clip reconstruction. Instead, definitive treatment of these complex aneurysms often involves proximal occlusion or trapping, and distal flow preservation through a revascularization bypass (Wessels et al., 2019; Xu et al., 2018). Such bypass can be challenging due to difficulty identifying the aneurysm's outflow and due to deep location of the recipient artery, which would require extensive subarachnoid dissection and a more demanding anastomosis. Thus, a few reports have described new techniques such as the flash fluorescence to simplify location of aneurysm's efferent territory and easily identify the most appropriate recipient artery for the revascularization bypass. The flash fluorescence technique consists of aneurysm dissection, temporary occlusion of one or several branches and after ICG administration, removal of the temporary clip. The efferent vessels will flash fluorescence on the cortical surface thus identifying a superficial recipient branch (Esposito et al., 2012; Rodriguez-Hernandez et al., 2012). When several vessels are deemed suitable, the vessel with the greatest caliber is chosen as the recipient. Alternatively, transdural ICG-VA (tICG-VA) has been proposed by Yokota et al. for non-selective cortical recipient vessel identification in revascularization procedures (Yokota et al., 2017). And a virtual planning method incorporating the ICG was described to aid in tailoring the approach over the recipient arteries (Dodier et al., 2020). However, despite their proven usefulness, all these strategies involve extensive aneurysm dissection, manipulation, and clipping of different branches to identify the cortical recipient artery with ICG-VA.

With the emergence of Hybrid OR, new treatment options have become available for challenging neurovascular lesions, and complex MCA aneurysms are not an exception. Hybrid operating rooms offer the possibility to combine the best of endovascular and microsurgical techniques. By merging techniques, Gruber and colleagues first described a “hybrid” flash fluorescence, with the injection of ICG proximal to a 10 mm MCA aneurysm in a young patient to identify the cortical recipient vessel for revascularization, after first having made selective angiograms to depict the cortical vessel anatomy and matching this with the morphology seen after intravenous ICG administration (Gmeiner et al., 2025; Gruber et al., 2012). We confirm the reproducibility of a similar technique described by Gruber et al. of a hybrid bypass procedure where we selectively identify the cortical recipient artery without manipulation of the aneurysm or its branches. In our three cases, we skipped the superselective angiogram comparison with regular ICG-VA and identified the appropriate recipient vessel by mimicking the microsurgical flash fluorescence technique with endovascular tools. This hybrid technique offers a less invasive way to identify the cortical recipient artery. It obviates the need for several microsurgical maneuvers such as Sylvian fissure opening, aneurysm trapping, temporary occlusion or distal branch dissection to find a suitable recipient artery. Altogether, the hybrid flash fluorescence may save valuable intraprocedural time and potentially decreases the risk of intraoperative rupture since the aneurysm is not manipulated.

Beyond the “hybrid flash fluorescence”, an H-OR opens further new perspectives and combined treatment options for complex intracranial aneurysms. As illustrated by our cases, the intraoperative angiography can aid not only in recipient vessel selection, but also in gold-standard evaluation of bypass patency and easier endovascular aneurysm trapping or PVO. Our three patients had their aneurysm occluded by microcoils, thus preventing the need for extensive dissection which may risk injury to eloquent brain or a potential aneurysm rupture. This microsurgical and endovascular combined treatment option is not new. Hacein-Bey et al. were among the first to report such combined approaches for a series of complex intracranial aneurysms. They described an STA-MCA bypass followed by endovascular PVO, albeit with aneurysm exposure during surgery and only performing endovascular PVO in a later stage (Hacein-Bey et al., 1998). The H-OR enables performing all treatment stages at the same place and in just one anesthesia time, which comes with several potential advantages. First, immediate aneurysm occlusion may prevent thrombosis of a bypass that has no real revascularization function until the aneurysm outflow is interrupted. Second, gold-standard in situ verification of the bypass patency allows easier and immediate correction on any technical mistakes regarding the anastomosis. Finally, saving the patient a second scheduled procedure may shorten the overall length of stay and may avoid the potential extra complications from a second anesthesia.

H-OR also offer many other relevant improvements for neurovascular pathologies, as described by a recent growing body of literature (Gmeiner et al., 2025; Gruber et al., 2012; Kerherve et al., 2025; Sato et al., 2018; Zhang et al., 2020a, 2020b; Choi et al., 2019; Jeon et al., 2019; Kim et al., 2020; Liao et al., 2019). As discussed and illustrated above, H-OR can be of great help for complex aneurysms requiring a revascularization bypass. But the H-OR can also aid in clip repositioning when intra-operative angiography shows parent vessel stenosis or aneurysm remnant, which is especially relevant when treating giant or morphologically complex aneurysms where microsurgical assessment of the clip position is not always possible (Sato et al., 2018; Zhang et al., 2020a). In giant aneurysms, endovascular parent vessel balloon occlusion or retrograde suction decompression can soften the aneurysm sac to facilitate clip placement (Hu et al., 2015). In the case of inadvertent premature intraoperative aneurysm rupture, the endovascular route can provide an elegant solution to gain intraluminal proximal control and diminish bleeding, aiding the surgical team to manage the procedure with greater ease (ref). In microsurgical AVM treatment, the intraoperative angiography enabled by the H-OR provides gold-standard control, and surgery can immediately be continued should a remnant be found (Choi et al., 2019; Jeon et al., 2019; Murayama et al., 2006). Even for patients in prone position e.g. for the treatment of spinal dAVF, immediate angiographic control using the popliteal artery access, enables to verify the complete obliteration of the fistula (Zhang et al., 2020b; Villelli et al., 2018). In aneurysmal subarachnoid hemorrhage cases, an H-OR can save time and patient transport. Furthermore, angiography performed after surgical treatment can diagnose vasospasm and subsequent treatment with intraarterial vasodilators or balloon angioplasty can be performed without any delay. Finally, rarer but nonetheless valuable treatment options enabled by a H-OR are the immediate surgical conversion in failed mechanical thrombectomy for ischemic stroke and surgical vascular access for endovascular catheters (e.g., direct vertebral artery, carotid artery or dural venous sinus puncture).

However, despite all the aforementioned advantages, H-ORs also come along with some drawbacks that may represent a further challenge for the treatment of complex MCA aneurysms (Gharios et al., 2023). First of all, H-ORs operating tables offer a quite limited range of motion which may difficult proper patient positioning, specially important when dealing with complex aneurysms morphology. Maintaining sterility during these hybrid procedures may also be cumbersome and could potentially worsen the outcome. Furthermore, a prolonged procedure time due to the learning curve of these hybrid new workflows may aggravate the sterility issue. Finally, a higher radiation exposure may be another concern of these procedures which is worth mentioning. Additionally endovascular techniques might induce thromboembolic complications. Nonetheless, further investigation and monitoring of these likely H-ORs limitations should help finding ways to overcome them and improve the hybrid techniques.

### 4.1 Limitations

The reader should be aware of a few limitations from our work worth mentioning. First and foremost, the case report character from the study curtails the sturdiness of our conclusions. Showing feasibility of the technique with a few cases is the first step, but larger cases series with control cohorts are necessary to draw solid conclusions about the safety and usefulness of the technique. Also, its important to consider the limited applicability of the technique as it stands now, since it is described for distal MCA aneurysms, which represent a scarce number of intracranial aneurysms. Finally, the availability of H-OR is limited due to its elevated cost, hence the described technique may not become common practice worldwide.

## 5 Conclusion

The hybrid combination technique using selective endovascular identification of the correct cortical artery for the STA-MCA bypass and subsequent endovascular occlusion of the parent artery was successful in treating complex distal fusiform MCA aneurysms in three patients. This technique provides a definitive treatment of the aneurysms while it avoids the risks of a large craniotomy, Sylvian fissure dissection and aneurysm manipulation. The hybrid operating room can result in proficient and safe treatment of different neurovascular pathologies. New and creative treatment solutions aimed at better patient outcomes will undoubtedly be found when treatment teams have this modality at their disposal.

## Declaration of competing interest

The authors declare that they have no known competing financial interests or personal relationships that could have appeared to influence the work reported in this paper.
